# 循环肿瘤DNA检测嵌合抗原受体T细胞治疗弥漫大B细胞淋巴瘤患者基因突变的可行性及其预后预测价值分析

**DOI:** 10.3760/cma.j.issn.0253-2727.2023.10.003

**Published:** 2023-10

**Authors:** 凌辉 周, 友琴 冯, 永仙 胡, 河 黄

**Affiliations:** 浙江大学医学院附属第一医院骨髓移植中心、浙江大学医学中心良渚实验室、浙江大学血液学研究所、浙江省干细胞与细胞免疫治疗工程实验室，杭州 310003 Bone Marrow Transplantation Center, the First Affiliated Hospital, Zhejiang University School of Medicine; Liangzhu Laboratory, Zhejiang University Medical Center; Institute of Hematology, Zhejiang University; Zhejiang Province Engineering Laboratory for Stem Cell and Immunity Therapy, Hangzhou 310003, China

**Keywords:** 弥漫大B细胞淋巴瘤, 嵌合抗原受体T细胞, 预后, 循环肿瘤DNA, Diffuse large B-cell lymphoma, Chimeric antigen receptor T-cell, Prognosis, ctDNA

## Abstract

**目的:**

探讨循环肿瘤DNA（ctDNA）检测在嵌合抗原受体T细胞（CAR-T细胞）治疗难治复发弥漫大B细胞淋巴瘤（R/R DLBCL）中的预后预测价值，为CAR-T细胞治疗失败患者的预防和后续治疗提供一定指导。

**方法:**

纳入2017年12月至2022年3月在浙江大学医学院附属第一医院接受CAR-T细胞治疗的48例R/R DLBCL患者。对患者治疗前外周血进行187个淋巴瘤相关基因集的ctDNA检测。将患者分为完全缓解（CR）和未达完全缓解（nonCR）两组。使用卡方检验和*t*检验比较组间临床特征的差异，使用Log-rank检验比较组间生存差异。

**结果:**

CAR-T细胞治疗R/R DLBCL nonCR患者中，突变频率最高的10个基因由高到低依次为TP53（41％）、TTN（36％）、BCR（27％）、KMT2D（27％）、IGLL5（23％）、KMT2C（23％）、MYD88（23％）、BTG2（18％）、MUC16（18％）、SGK1（18％）。Kaplan-Meier生存分析结果表明相较于ctDNA突变基因数≤10的患者，ctDNA突变基因数>10的患者总生存（OS）（1年OS率：0对73.8％，*P*<0.001）和无进展生存（PFS）较差（1年PFS率：0对51.8％, *P*＝0.011）。治疗前MUC16突变阳性的患者OS更好（2年OS率：56.8％对26.7％，*P*＝0.046），而BTG2突变阳性的患者OS较差（1年OS率：0对72.5％，*P*＝0.005）。

**结论:**

ctDNA检测可以作为评估CAR-T细胞治疗R/R DLBCL患者疗效的工具，治疗前的基因突变负荷、MUC16以及BTG2的突变具有潜在的预后预测价值。

## Abstract (en)

**Objective:**

To explore the prognostic value of circulating tumor DNA（ctDNA）testing in patients with refractory/relapsed diffuse large B-cell lymphoma（R/R DLBCL）undergoing chimeric antigen receptor T-cell（CAR-T）therapy, and to guide the prevention and subsequent treatment of CAR-T-cell therapy failure.

**Methods:**

In this study, 48 patients with R/R DLBCL who received CAR-T-cell therapy at the First Affiliated Hospital of Zhejiang University School of Medicine between December 2017 and March 2022 were included. Furthermore, ctDNA testing of 187 lymphoma-related gene sets was performed on peripheral blood samples obtained before treatment. The patients were divided into complete remission and noncomplete remission groups. The chi-square test and *t*-test were used to compare group differences, and the Log-rank test was used to compare the differences in survival.

**Results:**

Among the patients who did not achieve complete remission after CAR-T-cell therapy for R/R DLBCL, the top ten genes with the highest mutation frequencies were TP53（41％）, TTN（36％）, BCR（27％）, KMT2D（27％）, IGLL5（23％）, KMT2C（23％）, MYD88（23％）, BTG2（18％）, MUC16（18％）, and SGK1（18％）. Kaplan-Meier survival analysis revealed that patients with ctDNA mutation genes >10 had poorer overall survival（OS）rate（1-year OS rate: 0 *vs* 73.8％, *P*<0.001）and progression-free survival（PFS）rate（1-year PFS rate: 0 *vs* 51.8％, *P*＝0.011）compared with patients with ctDNA mutation genes ≤10. Moreover, patients with MUC16 mutation positivity before treatment had better OS（2-year OS rate: 56.8％ *vs* 26.7％, *P*＝0.046）, whereas patients with BTG2 mutation positivity had poorer OS（1-year OS rate: 0 *vs* 72.5％, *P*＝0.005）.

**Conclusion:**

ctDNA detection can serve as a tool for evaluating the efficacy of CAR-T-cell therapy in patients with R/R DLBCL. The pretreatment gene mutation burden, mutations in MUC16 and BTG2 have potential prognostic value.

CAR-T细胞疗法在治疗难治复发弥漫大B细胞淋巴瘤（R/R DLBCL）方面取得了巨大突破[1]–[4]。迄今为止，美国食品药品管理局（FDA）已分别于2017年8月、2017年10月、2021年2月批准了三种CAR-T细胞疗法用于DLBCL的治疗，分别为axicabtagene ciloleucel、tisagenelecleucel、lisocabtagene maraleucel。CAR-T细胞治疗通常是R/R DLBCL患者的为数不多的治疗选择。但是仍有近半的患者不能达到完全缓解（CR），这部分患者生存差，因此亟需寻找预测CAR-T细胞治疗R/R DLBCL 患者新的生物标志物，从而及早干预预后欠佳患者，改善生存。

循环肿瘤DNA（ctDNA）从细胞凋亡和（或）坏死的肿瘤细胞中释放，是淋巴瘤的一种新兴生物标志物[5]–[9]。从血液中提取ctDNA的便利性促进了肿瘤突变的鉴定和序列监测。与组织活检相比，外周血ctDNA检测显示出如下明显优势：无创或微创，可反复取材、收集、处理并且分析报告时间短；同时ctDNA能克服肿瘤空间异质性，可相对全面实时地反映患者的肿瘤分子特征；此外，ctDNA还可增加癌症驱动基因突变检出率[10]–[11]。目前ctDNA监测在CAR-T细胞治疗中的应用主要是通过免疫球蛋白重链（IgH）-VDJ、IgH-DJ和免疫球蛋白Kappa轻链或免疫球蛋白lambda轻链区域追踪克隆以评估微小残留[12]–[13]，然而，这种监测方法忽略了其他重要的遗传变异。探索更好的覆盖率和预后生物标志物的替代方法是必要的。

因此，本研究通过对接受CAR-T细胞治疗的DLBCL患者外周血进行187个淋巴瘤相关基因集ctDNA的检测，评估ctDNA监测在CAR-T细胞治疗R/R DLBCL患者预后中的应用价值，以预测患者的疾病转归并指导CAR-T细胞治疗失败患者的后续治疗。

## 病例与方法

一、患者纳入

本研究共收集48例2017年12月至2022年3月在本中心接受CAR-T细胞治疗的R/R DLBCL患者的治疗前血清样本进行187个淋巴瘤相关基因集的ctDNA检测。本研究通过浙江大学医学院附属第一医院伦理委员会批准（浙大一院伦审2023研第79号），所有患者均签署了样本获取知情同意书。

二、外周血血清的分离和制备

抽取患者外周血10 ml至EDTA抗凝管。使用PBS缓冲液以1∶1比例稀释样本，并用吸管混匀。按1∶2比例先将Ficoll溶液加入离心管，然后再用吸管将血样本沿管壁缓慢加至分离液上面，避免两者混合。在密度梯度离心机中进行离心，400 ×*g*离心25 min，加速度为4级，减速度为0级。离心结束后，得到上层血清，放至−80 °C冰箱保存，用于后续ctDNA测序。

三、ctDNA靶向文库制备

使用VAHTS Serum/Plasma Circulating DNA Kit（南京诺唯赞生物科技有限公司）从血清中提取DNA。利用VAHTS Universal DNA Library Prep Kit for lllumina V3（南京诺唯赞生物科技有限公司）建库。使用 Oubit 荧光定量（赛默飞世尔科技有限公司）和安捷伦 2200 TapeStation 系统质检。使用NextSeq 500（illumina）测序。

四、生物信息分析

原始图像数据文件，经过碱基识别分析转化为原始测序序列，结果以FASTQ文件格式存储，采取fastp软件对转化得到的FASTQ格式原始数据进行过滤去接头。使用BWA、samtools、PICARD、GATK等工具对过滤后的FASTQ格式文件进行比对、排序、标记重复、局部重比、质量值校验后得到校正后bam文件。使用samtools和VarScan2对比对校正后的bam文件进行变异检测，生成原始的vcf文件。使用ANNOVAR软件[14]对体细胞突变进行注释，添加相关字段。根据本地数据库和过滤条件对检出变异进行过滤，等级分类后汇总最终结果用于后续分析。使用maftools工具对后续肿瘤突变数据进行分析。根据突变对蛋白质结构的影响将突变分为：错义突变（missense mutation）、移码缺失（frameshift deletion），移码插入（frameshift insertion）、非移码缺失（nonframeshift deletion）、非移码插入（nonframeshift insertion）、无义突变（stopgain）、终止密码子缺失（stoplost）、启动密码子缺失（startloss）以及剪切突变（splicing）。

五、随访

采用电话或查阅患者病历的方式进行随访，随访截止时间为2022年9月。

六、统计学处理

研究的主要终点为CAR-T细胞治疗后R/R DLBCL患者的无进展生存（PFS）和总生存（OS）。PFS期被定义为从接受CAR-T细胞回输至出现肿瘤进展或死亡的时间间隔；OS期被定义为从接受CAR-T细胞回输至因任何原因引起死亡的时间间隔。采用Kaplan-Meier法绘制生存曲线，采用Log-rank检验比较组间的生存事件差异。使用卡方检验或Fisher精确检验对分类变量进行差异分析。所有统计分析均采用R软件（version 4.0.3）进行，*P*<0.05为差异具有统计学意义。

## 结果

一、患者临床特征

本研究回顾性分析了自2017年12月至2022年3月在本中心接受CAR-T细胞治疗的48例R/R DLBCL患者，其中26例达到CR，14例达到部分缓解（PR），8例疾病稳定（SD）或疾病进展（PD）。患者的基线特征如表1所示，CR组和未达CR（nonCR）组间的年龄、性别、分型、末次化疗间隔时间、化疗线数、是否经过移植、细胞因子释放综合征（CRS）等级、Ann Arbor分期、IPI评分以及基线乳酸脱氢酶和β_2_微球蛋白水平差异均无统计学意义。

**表1:** CAR-T细胞治疗后CR组与nonCR组弥漫大B细胞淋巴瘤患者临床特征比较

指标	总数（48例）	CR组（26例）	nonCR组（22例）	*P*值
性别［例（％）］				0.90
女	19（40）	11（42）	8（36）	
男	29（60）	15（58）	14（64）	
年龄［岁，*M*（*Q*_1_,*Q*_3_）］	59.5（46.75, 65.25）	60.5（49, 65.75）	54.5（43.25, 65）	0.40
分型				0.23
GCB	9（19）	6（23）	3（14）	
nonGCB	31（65）	14（54）	17（77）	
NA	8（16）	6（23）	2（9）	
末次化疗间隔时间［月，*M*（*Q*_1_,*Q*_3_）］	54（36, 102）	91.5（41.25, 108）	49（33, 92）	0.16
化疗线数［例（％）］				0.48
≤2线	15（33）	7（29）	8（36）	
>2线	33（67）	19（71）	14（64）	
是否移植［例（％）］				1.00
否	43（90）	23（88）	20（91）	
是	5（10）	3（12）	2（9）	
乳酸脱氢酶［U/L，*M*（*Q*_1_,*Q*_3_）］	286.00（225.75, 409.50）	279.50（229.75, 412.50）	297.50（231.00, 361.25）	0.90
β_2_微球蛋白［µg/L，*M*（*Q*_1_,*Q*_3_）］	2 595.0（1 952.5, 3 575.0）	2 809.5（2 037.5, 3 575.0）	2 460.0（1 977.5, 3 190.0）	0.82
Ann Arbor分期［例（％）］				0.56
Ⅰ/Ⅱ期	7（14）	5（19）	2（9）	
Ⅲ/Ⅳ期	41（86）	21（81）	20（91）	
IPI评分［例（％）］				0.32
0～1分	8（16）	6（23）	2（9）	
2～3分	32（67）	17（65）	15（68）	
4～5分	8（17）	3（12）	5（23）	
CRS等级［例（％）］				0.28
0～1级	28（59）	17（66）	11（50）	
2～3级	20（41）	9（35）	11（50）	

注 CR：完全缓解；nonCR：未达完全缓解；CRS：细胞因子释放综合征；GCB：生发中心来源；nonGCB：非生发中心来源；IPI：淋巴瘤的国际预后指数

二、CAR-T细胞治疗DLBCL患者基因突变图谱

对突变信息进行注释和过滤后，48个样本以错义突变为主（图1A、B），其中又以C>T和T>C突变为主（图1C），中位ctDNA突变数目为7个（图1D），错义突变的中位ctDNA最高（图1E）。如图1F所示，突变频率最高的10个基因由高到低依次为TTN（42％）、BCR（35％）、TP53（29％）、KMT2D（23％）、IGLL5（21％）、KMT2C（21％）、MUC16（19％）、DNMT3A（15％）、MYD88（15％）、PIM1（12％）。突变频率最高的15个基因的变异等位基因频率（VAF）分布如图1G所示。不同疗效组患者基因突变图谱如图2所示，CR组突变频率最高的10个基因由高到低依次为TTN（46％）、BCR（42％）、IGLL5（19％）、KMT2C（19％）、KMT2D（19％）、MUC16（19％）、TP53（19％）、NOTCH（15％）、BTG1（12％）、PIM1（12％）；在nonCR组中，突变频率最高的10个基因由高到低依次为TP53（41％）、TTN（36％）、BCR（27％）、KMT2D（27％）、IGLL5（23％）、KMT2C（23％）、MYD88（23％）、BTG2（18％）、MUC16（18％）、SGK1（18％）。

**图1:**
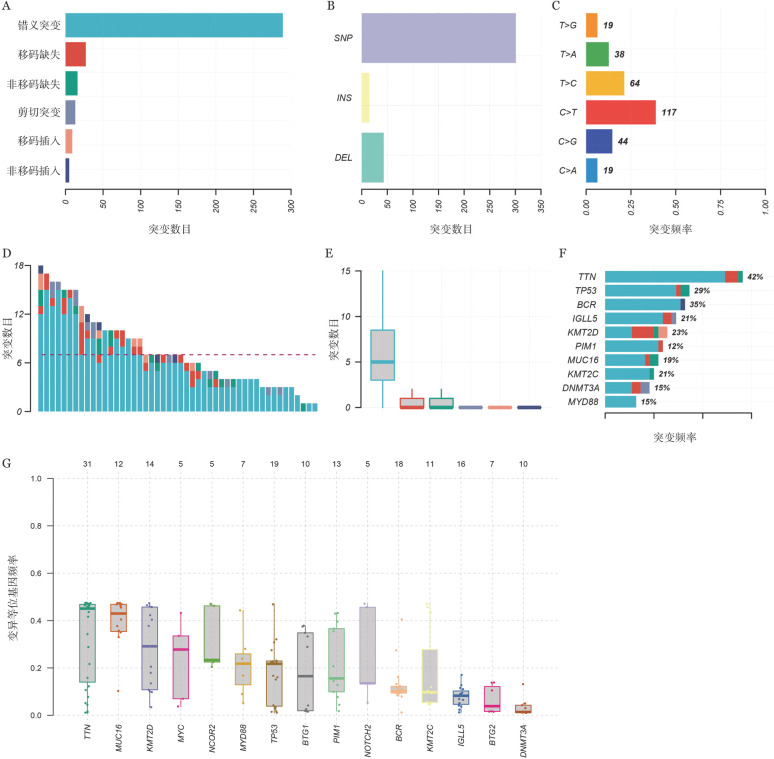
48例难治复发弥漫大B细胞淋巴瘤患者治疗前的血清样本的循环肿瘤DNA（ctDNA）突变景观图 A 6种主要突变数目柱状图； B 3种突变类型柱状图，SNP：点突变；DEL：缺失突变；INS：插入突变； C 6种SNV突变频谱； D 每个样本ctDNA突变数目柱状图； E 所有样本6种主要突变数目箱型图，从左往右分别代表错义突变、移码缺失、非移码缺失、剪切突变、移码插入、非移码插入； F 突变频率前10基因的突变频率； G 突变频率最高的15个基因的变异等位基因频率分布

**图2:**
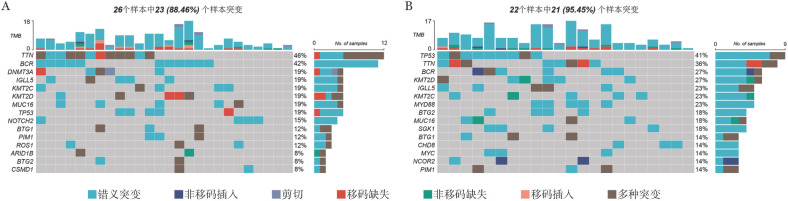
完全缓解组（A）与未达完全缓解组（B）循环肿瘤DNA突变瀑布图

将CR组和nonCR组患者治疗前的特定基因分配到每个通路上，评估了10条与癌症研究相关的典型信号通路，包括细胞周期、Hippo、Myc、Notch、Nrf2、PI3K、RTK-RAS、TGFb、p53和Wnt通路，结果表明CR组受影响频率最高的三条信号通路由高到低依次为RTK-RAS（38.5％）、TP53（19.2％）、NOTCH（19.2％）信号通路，而nonCR组受影响频率最高的三条信号通路由高到低依次为TP53（45.5％）、RTK-RAS（36.4％）、NOTCH（31.8％）信号通路（图3）。

**图3:**
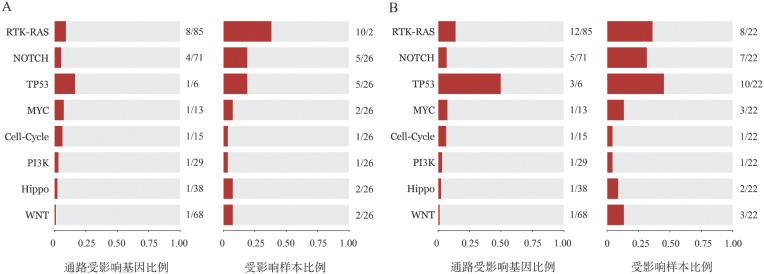
完全缓解组（A）与未达完全缓解组（B）致癌生物途径的富集

三、CAR-T细胞治疗DLBCL患者基因突变的互斥性与共现性

Oncodrive函数结果表明NCOR2、MYD88和BCR可能是接受CAR-T细胞疗法的R/R DLBCL患者的癌症驱动基因（图4A）。随后用pfamDomains函数来注释和统计导致氨基酸改变的突变所在pfam结构域，结果表明出现突变频率最高的5个pfam结构域由高到低依次为FN3、P53、RhoGAP_Bcr、Pkinase以及I-set（图4B）。此外，通过somaticInteractions函数对基线ctDNA突变频率最高的15个基因两两之间进行成对的Fisher精确检验分析突变的互斥性和共现性，共发现4对共现性的基因对（*P*<0.05），未发现互斥性基因对。其中，MYD88与BTG2以及IGLL5呈共现性，BCR与MYC呈共现性，而BTG1与PIM1呈共现性，具体结果如图4C所示。

**图4:**
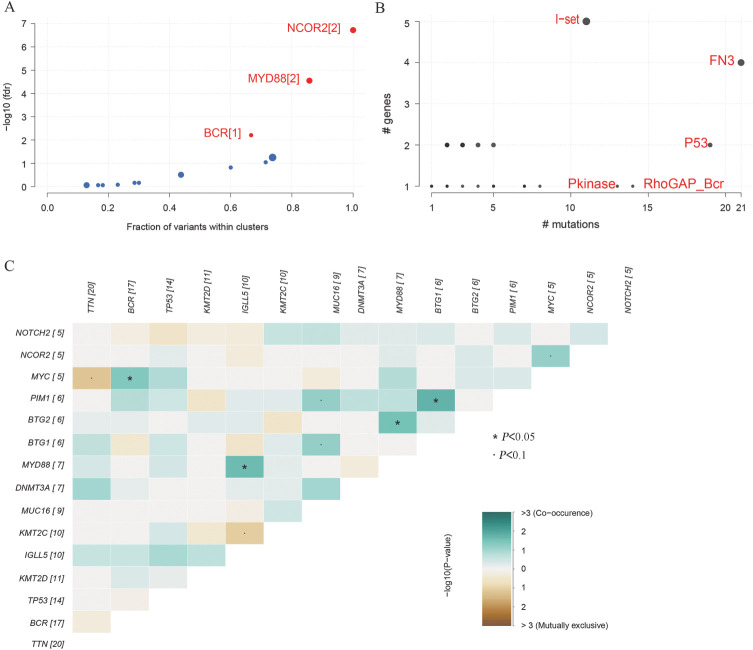
循环肿瘤DNA（ctDNA）突变的互斥性与共现性 A 基线时由 oncodrive 鉴定的疾病相关驱动基因（FDR<0.05），图中点的大小以及基因名后的数字展示的是基因内突变cluster的数量，X轴为cluster内的突变数量或者占突变总数的比例，Y轴是-log10(FDR)； B Pfam蛋白结构域和基因数散点图，圆点代表pfam结构域，对应X轴为该结构域中出现的突变数量，Y轴以及圆点的大小为影响的基因数，参数top用来选择标记突变数最多的pfam domain数量； C 基线时中互斥和共存的ctDNA对显示为三角矩阵。绿色表示共现趋势，而黄色表示排它性趋势

四、ctDNA在CAR-T细胞治疗DLBCL预后中的作用

根据每例患者检测到的突变基因的数目将患者分为≤10个和>10个突变基因的两组，治疗前突变基因>10个的患者OS（1年OS率：0对73.8％，*P*<0.001）（图5A）和PFS（1年PFS率：0对51.8％，*P*＝0.011）（图5B）更差。对治疗前突变频率大于10％的突变基因进行Kaplan-Meier生存分析，结果表明治疗前MUC16突变阳性的患者OS更好（2年OS率：56.8％对26.7％，*P*＝0.046）（图5C），而BTG2突变阳性的患者OS较差（1年OS率：0对72.5％，*P*＝0.005）（图5D）。

**图5:**
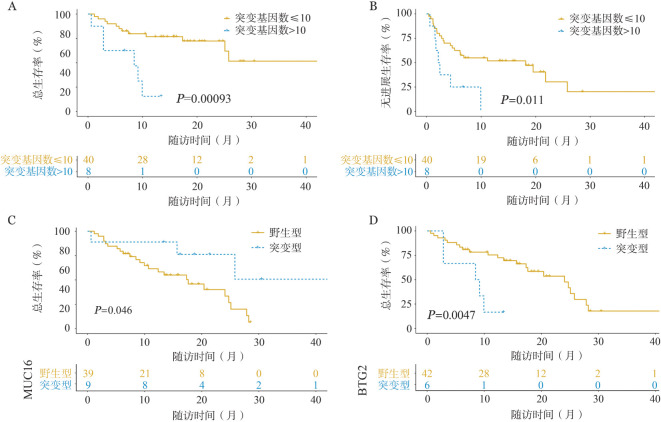
循环肿瘤DNA（ctDNA）对CAR-T细胞治疗弥漫大B细胞淋巴瘤患者预后的预测作用 A 治疗前不同ctDNA突变基因数目组的总生存曲线； B 治疗前不同ctDNA突变基因数目组的无进展生存曲线； C 治疗前不同MUC16状态组的总生存曲线； D 治疗前不同BTG2状态组的总生存曲线

## 讨论

尽管在大量的R/R DLBCL患者的临床实践中，CAR-T细胞疗法展现了前所未有的有效性，但仍有近半的患者不能缓解或缓解后出现复发，这部分患者往往后续可选治疗方案有限，生存期短[15]–[16]。因此，提前预测CAR-T细胞治疗疗效欠佳的患者以制定早期干预方案至关重要。

癌症基因组测序计划加深了对不同癌种基因组突变谱的理解，发现了很多新的癌症驱动基因突变以及疗效预测标志物。然而，对于接受CAR-T细胞治疗的R/R DLBCL患者的基因突变谱及其分子生物学的研究仍然有限。R/R DLBCL研究进展受到的最主要限制是DLBCL组织标本的获取困难，穿刺活检样本往往难以满足检测要求，并且深层组织部位的DLBCL样本难以获得。因此，在临床上迫切需要寻找一种能够替代穿刺组织活检的标本，全面实时地反映DLBCL患者肿瘤分子特征。ctDNA的预后预测作用已经得到很好的证实，治疗前和治疗中的浓度都能很好地评估CAR-T细胞治疗的预后[13],[17]。我们前期的一项8例动态ctDNA监测的研究结果表明了类似的结果，治疗前ctDNA的阳性突变基因数目与接受CAR-T细胞治疗的DLBCL患者的长期预后有关[18]。本研究共入组了48例接受CAR-T细胞治疗的R/R DLBCL患者，对治疗前外周血进行187个淋巴瘤相关基因集的捕获和测序，绘制了接受CAR-T细胞治疗的难治复发DLBCL患者的基因突变图谱，并分析了不同疗效组基因突变图谱的差异、癌症相关通路的差异以及其在预后预测中的作用。进一步证实了治疗前ctDNA的阳性突变基因数目能很好地预测CAR-T治疗DLBCL患者的临床转归。

此外，研究发现治疗前MUC16（CA125）以及BTG2两个基因突变对CAR-T细胞治疗DLBCL患者的预后具有预测作用。MUC16是一种Ⅰ型跨膜黏蛋白，由C末端结构域、串联重复区和细胞外N末端三部分组成，已被广泛用作卵巢癌的生物标志物，其表达与疾病进展有关[19]。多项研究表明，MUC16在多种癌症类型中过度表达，并且，其过表达与多种恶性肿瘤预后较差有关[20]–[23]。一项大样本胃癌基因组测序结果表明：MUC16突变可能与更好的预后以及免疫反应和细胞周期信号通路有关[24]。另一项对黑色素瘤的基因组研究同样显示了类似的结果：MUC16突变可能会影响免疫相关途径和肿瘤浸润免疫细胞亚群，从而改善黑色素瘤患者的预后[25]。目前尚未见MUC16在CAR-T细胞治疗中的报道。本研究结果表明治疗前MUC16突变阳性的患者相较于MUC16野生型患者预后更好。这提示MUC16可能是CAR-T细胞治疗DLBCL的潜在生物标志物。BTG/TOB基因家族是一个抗增殖基因家族，被发现在调节细胞周期、凋亡和分化等方面都发挥着重要作用。BTG2作为该家族第一个被发现的基因,已被证实在淋巴恶性肿瘤和实体瘤中发挥肿瘤抑制因子的作用[26]。之前多项全基因组分析结果研究表明，在B细胞恶性肿瘤中经常观察到BTG2的遗传畸变[27]–[28]，这表明BTG2的突变可能导致其肿瘤抑制因子作用减弱从而导致肿瘤的发生发展。在本研究中12％的DLBCL患者发生了BTG2的突变，并且BTG2突变阳性的患者OS较差。这提示BTG2突变可能可以作为CAR-T细胞治疗DLBCL患者预后差的标志。

总体而言，本研究探索了ctDNA在CAR-T细胞治疗R/R DLBCL患者中作为预后生物标志物的可行性。治疗前的基因突变个数、MUC16以及BTG2突变状态在CAR-T细胞疗法中具有潜在的预后价值，但本研究的样本量仍较为有限，研究结果需要在更大的独立队列中进行验证。

## References

[1] Kochenderfer JN, Dudley ME, Kassim SH (2015). Chemotherapy-refractory diffuse large B-cell lymphoma and indolent B-cell malignancies can be effectively treated with autologous T cells expressing an anti-CD19 chimeric antigen receptor[J]. J Clin Oncol.

[2] Kochenderfer JN, Somerville R, Lu T (2017). Lymphoma Remissions Caused by Anti-CD19 Chimeric Antigen Receptor T Cells Are Associated With High Serum Interleukin-15 Levels[J]. J Clin Oncol.

[3] Locke FL, Neelapu SS, Bartlett NL (2017). Phase 1 Results of ZUMA-1: A Multicenter Study of KTE-C19 Anti-CD19 CAR T Cell Therapy in Refractory Aggressive Lymphoma[J]. Mol Ther.

[4] Neelapu SS, Locke FL, Bartlett NL (2017). Axicabtagene Ciloleucel CAR T-Cell Therapy in Refractory Large B-Cell Lymphoma[J]. N Engl J Med.

[5] Kurtz DM, Green MR, Bratman SV (2015). Noninvasive monitoring of diffuse large B-cell lymphoma by immunoglobulin high-throughput sequencing[J]. Blood.

[6] Roschewski M, Dunleavy K, Pittaluga S (2015). Circulating tumour DNA and CT monitoring in patients with untreated diffuse large B-cell lymphoma: a correlative biomarker study[J]. Lancet Oncol.

[7] Scherer F, Kurtz DM, Newman AM (2016). Distinct biological subtypes and patterns of genome evolution in lymphoma revealed by circulating tumor DNA[J]. Sci Transl Med.

[8] Rossi D, Diop F, Spaccarotella E (2017). Diffuse large B-cell lymphoma genotyping on the liquid biopsy[J]. Blood.

[9] 宋 妮妮, 盛 立霞, 欧阳 桂芳 (2019). 循环肿瘤DNA在B细胞非霍奇金淋巴瘤中的研究进展[J]. 白血病·淋巴瘤.

[10] Diaz LA, Bardelli A (2014). Liquid biopsies: genotyping circulating tumor DNA[J]. J Clin Oncol.

[11] Crowley E, Di Nicolantonio F, Loupakis F (2013). Liquid biopsy: monitoring cancer-genetics in the blood[J]. Nat Rev Clin Oncol.

[12] Hossain NM, Dahiya S, Le R (2019). Circulating tumor DNA assessment in patients with diffuse large B-cell lymphoma following CAR T-cell therapy[J]. Leuk Lymphoma.

[13] Frank MJ, Hossain NM, Bukhari A (2021). Monitoring of Circulating Tumor DNA Improves Early Relapse Detection After Axicabtagene Ciloleucel Infusion in Large B-Cell Lymphoma: Results of a Prospective Multi-Institutional Trial[J]. J Clin Oncol.

[14] Wang K, Li M, Hakonarson H (2010). ANNOVAR: functional annotation of genetic variants from high-throughput sequencing data[J]. Nucleic Acids Res.

[15] 张 琪, 肖 毅 (2022). CD19嵌合抗原受体T细胞治疗弥漫大B细胞淋巴瘤的复发机制及应对策略[J]. 中华血液学杂志.

[16] 肖 霞, 江 嫣雨, 曹 雅青 (2019). CD19CAR-T细胞治疗B细胞淋巴瘤22例疗效及安全性[J]. 中华血液学杂志.

[17] Sworder BJ, Kurtz DM, Alig SK (2023). Determinants of resistance to engineered T cell therapies targeting CD19 in large B cell lymphomas[J]. Cancer Cell.

[18] Zhou L, Zhao H, Shao Y (2021). Serial surveillance by circulating tumor DNA profiling after chimeric antigen receptor T therapy for the guidance of r/r diffuse large B cell lymphoma precise treatment[J]. J Cancer.

[19] Felder M, Kapur A, Gonzalez-Bosquet J (2014). MUC16 (CA125): tumor biomarker to cancer therapy, a work in progress[J]. Mol Cancer.

[20] Shimizu A, Hirono S, Tani M (2012). Coexpression of MUC16 and mesothelin is related to the invasion process in pancreatic ductal adenocarcinoma[J]. Cancer Sci.

[21] Chen SH, Hung WC, Wang P (2013). Mesothelin binding to CA125/MUC16 promotes pancreatic cancer cell motility and invasion via MMP-7 activation[J]. Sci Rep.

[22] Higashi M, Yamada N, Yokoyama S (2012). Pathobiological implications of MUC16/CA125 expression in intrahepatic cholangiocarcinoma-mass forming type[J]. Pathobiology.

[23] Liang C, Qin Y, Zhang B (2017). Oncogenic KRAS Targets MUC16/CA125 in Pancreatic Ductal Adenocarcinoma[J]. Mol Cancer Res.

[24] Li X, Pasche B, Zhang W (2018). Association of MUC16 Mutation With Tumor Mutation Load and Outcomes in Patients With Gastric Cancer[J]. JAMA Oncol.

[25] Wang Z, Hou H, Zhang H (2022). Effect of MUC16 mutations on tumor mutation burden and its potential prognostic significance for cutaneous melanoma[J]. Am J Transl Res.

[26] Kim SH, Jung IR, Hwang SS (2022). Emerging role of anti-proliferative protein BTG1 and BTG2[J]. BMB Rep.

[27] Lohr JG, Stojanov P, Lawrence MS (2012). Discovery and prioritization of somatic mutations in diffuse large B-cell lymphoma (DLBCL) by whole-exome sequencing[J]. Proc Natl Acad Sci U S A.

[28] Morin RD, Mendez-Lago M, Mungall AJ (2011). Frequent mutation of histone-modifying genes in non-Hodgkin lymphoma[J]. Nature.

